# Probiotic Mixture Containing *Lactobacillus helveticus*, *Bifidobacterium longum* and *Lactiplantibacillus plantarum* Affects Brain Responses to an Arithmetic Stress Task in Healthy Subjects: A Randomised Clinical Trial and Proof-of-Concept Study

**DOI:** 10.3390/nu14071329

**Published:** 2022-03-22

**Authors:** Hanna M. T. Edebol Carlman, Julia Rode, Julia König, Dirk Repsilber, Ashley N. Hutchinson, Per Thunberg, Jonas Persson, Andrey Kiselev, Jens C. Pruessner, Robert J. Brummer

**Affiliations:** 1 Nutrition-Gut-Brain Interactions Research Centre, Faculty of Medicine and Health, School of Medical Sciences, Örebro University, 70182 Örebro, Sweden; hmtcarlman@gmail.com (H.M.T.E.C.); julia.konig@oru.se (J.K.); dirk.repsilber@oru.se (D.R.); ashley.hutchinson@oru.se (A.N.H.); robert.brummer@oru.se (R.J.B.); 2 Department of Radiology and Medical Physics, Faculty of Medicine and Health, School of Medical Sciences, Örebro University, 70182 Örebro, Sweden; per.thunberg@regionorebrolan.se; 3 Center for Lifespan Developmental Research (LEADER), Faculty of Humanities and Social Sciences, School of Law, Psychology and Social Work, Örebro University, 70182 Örebro, Sweden; jonas.persson@oru.se; 4 Center for Applied Autonomous Sensor Systems, Faculty for Business, Science and Engineering, School of Natural Science and Technology, Örebro University, 70182 Örebro, Sweden; andrey.kiselev@oru.se; 5 Douglas Institute, McGill University, Montréal, QC H4H1R3, Canada; jens.pruessner@uni-konstanz.de; 6 Department of Psychology, University of Konstanz, 78457 Konstanz, Germany

**Keywords:** functional magnetic resonance imaging (fMRI), brain activity, gut microbiota, Montreal Imaging Stress Task (MIST), autonomic nervous system, gut-brain axis

## Abstract

Probiotics are suggested to impact physiological and psychological stress responses by acting on the gut-brain axis. We investigated if a probiotic product containing *Bifidobacterium longum* R0175, *Lactobacillus helveticus* R0052 and *Lactiplantibacillus plantarum* R1012 affected stress processing in a double-blinded, randomised, placebo-controlled, crossover proof-of-concept study (NCT03615651). Twenty-two healthy subjects (24.2 ± 3.4 years, 6 men/16 women) underwent a probiotic and placebo intervention for 4 weeks each, separated by a 4-week washout period. Subjects were examined by functional magnetic resonance imaging while performing the Montreal Imaging Stress Task (MIST) as well as an autonomic nervous system function assessment during the Stroop task. Reduced activation in regions of the lateral orbital and ventral cingulate gyri was observed after probiotic intervention compared to placebo. Significantly increased functional connectivity was found between the upper limbic region and medioventral area. Interestingly, probiotic intervention seemed to predominantly affect the initial stress response. Salivary cortisol secretion during the task was not altered. Probiotic intervention did not affect cognitive performance and autonomic nervous system function during Stroop. The probiotic intervention was able to subtly alter brain activity and functional connectivity in regions known to regulate emotion and stress responses. These findings support the potential of probiotics as a non-pharmaceutical treatment modality for stress-related disorders.

## 1. Introduction

Physiological and psychological stress can affect gut function and its interaction with the brain, the so-called gut-brain axis. The main components of the psychobiology of stress are the hypothalamic-pituitary-adrenal axis (HPA axis), the sympathetic-adreno-medullary axis and the sympatho-neural systems, all of which regulate the secretion of gluco- corticoids (e.g., cortisol) and catecholamines into the bloodstream [1]. The HPA axis is an essential component of communication between the brain and the gut [2]. It is a major neuroendocrine system that regulates reactions to both chronic and acute stress. For example, acute and chronic mental stress are known to damage the integrity of the gut epithelium (by corticotropin-releasing hormone-induced mast cell destabilisation and degranulation) [3] and to alter the habitat for bacteria [4,5,6]. This in turn may result in altered gut microbiota composition and activity, potentially having an influence on brain function. Furthermore, stressful life events can impact the development and course of disorders of gut-brain interactions such as irritable bowel syndrome [7]. 

Manipulation of the gut microbiota using probiotic bacteria (living microorganisms which, if consumed in adequate amounts, confer a health effect on the host [8]) has been shown to improve cognition and stress response in preclinical [9,10,11,12,13,14] and clinical studies [15,16,17,18,19]. However, only a few of those studies have specifically investigated brain responses to acute stressors or cognitive tasks [17,18]. A four-week intervention with a multi-strain probiotic containing nine bacterial strains belonging to *Lactobacillus*, *Lactococcus* and *Bifidobacterium* species had no effect on reaction time and brain response patterns during neurocognitive tasks such as an emotional face-matching paradigm, an emotional face-word Stroop paradigm and the classic colour-word Stroop paradigm in healthy subjects, but positively affected cognitive task performance when combined with an acute stress challenge [17]. Alterations in brain activity (using electroencephalography [EEG]) during a social stress task (cyberball game) following a four-week administration of *Bifidobacterium longum* in healthy subjects were reported in another study [18]. These last two studies would suggest that probiotics do not affect cognitive abilities per se, but may have an effect on cognitive tasks in the presence of stress. Thus, for the current study, we aimed to combine a probiotic intervention with an investigation of gut-brain axis signalling, with a focus on the HPA axis and the autonomic nervous system (ANS), which are important signalling routes in the communication between the gut and the brain. Experimentally, we applied a stressor to challenge brain-gut signaling and assessed the response on the level of autonomic and central nervous system function. Such an approach might allow for investigating the relationship between brain function and alterations in the gut microbiota.

Probiotic interventions are likely to have different effects depending on the strains used. Here, we used a probiotic mixture of *Bifidobacterium longum* R0175, *Lactobacillus helveticus* R0052 and *Lactiplantibacillus plantarum* R1012 (formerly known as *Lactobacillus plantarum*). The administration of this probiotic mixture has been shown to partially reverse negative behavioural changes in a mouse model of chronic mild stress [20], which supports the use of this probiotic mixture in a human study.

Different types of stress and different phases of stress regulation can affect the brain in different ways. To understand the role of probiotics on brain function in stress regulation, it is important to apply different stressors which evoke different brain circuitry. Thus, we utilised a well-validated arithmetic stressor, the Montreal Imaging Stress Task (MIST) [21], during brain imaging for the first time in the context of a probiotic intervention study. In contrast to previously applied stressors used in probiotic studies, this stressor is a combination of mental arithmetic and negative social evaluation based on the Trier social stress test [21,22]. The MIST paradigm has been shown to evoke task-related brain activity changes in brain regions implicated in the stress response, emotional regulation and cognition, including the occipital cortex, prefrontal cortex, cingulate cortex, and limbic regions, amongst others [23].

Taken together, the present proof-of-concept study investigated if a probiotic product containing *Bifidobacterium longum* R0175, *Lactobacillus helveticus* R0052 and *Lactiplantibacillus plantarum* R1012 (a total of 3 × 10^9^ colony forming units, CFU, per day at the end of shelf life, i.e., number of viable bacteria) affected functional brain responses in healthy subjects during an arithmetic stress task (the MIST). Brain response changes were assessed using functional magnetic resonance imaging (fMRI). We hypothesised that the probiotic intervention dampens stress responses and thus affects the activity of brain regions implicated in the different phases of stress, i.e., limbic regions as well as regions of the prefrontal and orbitofrontal cortices. Additionally, the Stroop task was used as a cognitive stressor; cognitive performance as well as autonomic nervous system activity were assessed during this task. Moreover, additional markers of stress such as salivary cortisol and self-ratings during the MIST were analysed.

## 2. Materials and Methods

### 2.1. Design

A randomised double-blinded placebo-controlled crossover study with 22 healthy subjects was performed in order to assess the effects of a probiotic intervention on brain activity and stress response. The primary outcome parameter was the effect of probiotic intervention on brain response patterns during an emotional attention task, which is reported separately (not yet published). The secondary endpoint was to detect differences in brain response patterns during the MIST linked to the probiotic intervention when compared to a placebo. Exploratory outcome parameters included the effect of the probiotic intervention on stress during the MIST (assessed by salivary cortisol and self-rated questionnaires) and on cognitive ability and autonomic nervous system function during a stressful condition (the Stroop test), amongst others.

**Figure 1:**
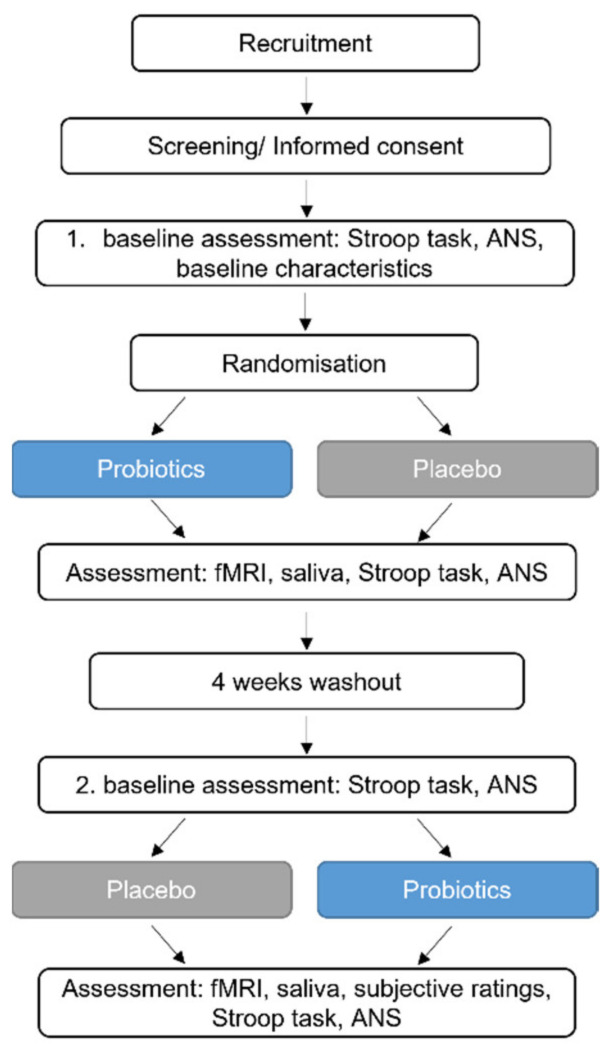
Study design; ANS—Autonomic nervous system, fMRI—Functional magnetic resonance imaging.

After baseline assessments, study participants received the probiotics or the placebo for four weeks, followed by a four-week washout period and a subsequent four-week intervention period (placebo or probiotics, respectively). The MIST was performed during fMRI at the end of each intervention period, but not at baseline to counteract a potential learning effect caused by repetitive testing. At several timepoints during the fMRI investigations, saliva samples were collected. Before and after the first and second intervention period, the Stroop test was performed together with an autonomic nervous system measurement. The study design is presented in Figure 1.

### 2.2. Subjects

Subjects were recruited by advertisement at Örebro University in 2018. Sample size calculation was based on the primary outcome, which is reported separately (not published yet). Details can be found in the Supplementary Materials. Inclusion criteria were 18–65 years of age and a signed informed consent. Exclusion criteria were chosen in order to recruit a healthy study population. A list of the exclusion criteria can be found in Supplementary Table S1. Participants were instructed to keep their diet, medication and lifestyle stable throughout the study. A participant flow chart can be found in Supplementary Figure S1.

### 2.3. Study Intervention

The probiotic product (a total of 3 × 10^9^ CFU at the end of shelf life), commercially available in Italy (named Puraflor, GSK Consumer Healthcare, Milano, Italy), was manufactured by a third party manufacturer, SIIT S.r.l. (Milano, Italy), and contained a combination of the three probiotic strains: *Lactobacillus helveticus* R0052 (CNCM-I-1722; 2 × 10^9^ CFU), *Lactiplantibacillus plantarum* R1012 (CNCM-I-3736; 8 × 10^8^ CFU), and *Bifidobacterium longum* R0175 (CNCM-I-3470; 7 × 10^7^ CFU), in addition to inulin, zinc, magnesium, potassium, glutathione and lactoferrin (Supplementary Table S2). The total daily amount of the probiotic strains in the product was a minimum of 3 × 10^9^ CFU per 3 g powder sachet (at the end of shelf life). Study participants were instructed to consume one sachet per day immediately after dissolving it in a glass of water and together with breakfast. Subjects recorded the daily intake of the probiotics and returned any unused sachets at the end of each intervention period for the purpose of measuring compliance (Supplementary Tables S3 and S4). 

The placebo product was a 2 g powder sachet without the bioactive compounds. In order to provide similar appearance and taste, both the probiotic product and the placebo contained sweeteners including fructose, acesulfame K and sucralose as well as orange flavour, citric acid, anticaking agent silicon dioxide and colouring. 

### 2.4. Randomisation and Masking

Subjects were randomised to two study groups receiving the intervention in different orders (probiotics or placebo first). The order of administration of the intervention (probiotics or placebo first) was carried out using a computerised randomisation list, and block randomisation with a random block size of six and four was applied. Half of the study group received the probiotics first and the placebo second, and vice versa for the other half of the study group. The assignment of administration order to the two groups and labelling of the sachets with subject ID and number of intervention periods was performed by a university staff member not involved in the study. The randomisation key was controlled by this person and not revealed until analysis of the primary outcome parameter was finished. The study was blinded for participants and study staff.

### 2.5. fMRI Protocol

Brain activity during task performance was measured using fMRI after four weeks of probiotic and placebo intervention, respectively. All fMRI acquisitions were performed with the same protocol with an initial 4.5-min structural scan, followed by a 5-min eyes-closed resting scan (not included in the current analysis), and an emotional attention task (not included in the current analysis) followed by the MIST paradigm. All fMRI acquisitions were performed with the same protocol, implying an equal sequential order for all acquisitions. A 3.0T MR system (Discovery 750 w, GE Medical Systems, WI) and a 32 channel fMRI head coil were used for all MR examinations. The structural scan (T1w IR-prepared fast spoiled gradient recalled echo, “BRAVO”) had the following parameters applied; TR/TE = 8.6/3.3 ms, acquired voxel size of 0.9 × 0.9 × 1.2 mm^3^, while the fMRI acquisitions were based on a gradient echo EPI pulse sequence using the following parameters: TR/TE = 2500/35 ms, slice thickness of 3.6 mm, no slice gap, in-plane resolution of 3.75 × 3.75 × 3.6 mm^3^ and a reduction factor (ASSET) of 2.

Subjects were instructed not to perform physical exercise and not to drink more than one cup of coffee or tea in the morning of the day of the fMRI assessment. Furthermore, subjects were encouraged to follow the same routines on both fMRI examination days. 

### 2.6. The Montreal Imaging Stress Task, MIST

Psychological stress was induced with the MIST as previously described [21,23]. The MIST was performed in three runs (7:05 min each), each run including six blocks consisting of a rest, control and experimental condition, always in the same order (rest-control-experimental-rest-control-experimental). A scheme of the MIST paradigm can be found in Supplementary Figure S2. During the experimental condition, subjects were exposed to challenging mental arithmetic tasks presented on a computer screen and were asked to respond by choosing a one-digit number using a rotary dial managed by hand-held response grips with four buttons. The difficulty and time limit of the tasks were manipulated automatically by the program’s algorithm adapting to the user performance by being just beyond each subject’s cognitive capacity and thus to result in a 20–45% performance range. A mock performance indicator suggested poor performance on the part of the subject in comparison to the average user, and additional negative feedback was provided both by the program (after incorrect or timed out answers) and verbally by the investigator (between runs). In the control condition, subjects were presented with the mental arithmetic task without time constraints and negative feedback. During the rest condition, the user interface was presented without arithmetic tasks.

Subjects were debriefed after their last study visit that the task was designed to be beyond their mental capacity and not meant to measure their ability to perform mental arithmetic.

### 2.7. Saliva Sample Collection and Analysis

Saliva samples were collected using Salivette collection tubes (Sarstedt, Germany) for measurement of cortisol at six time points during each of the two fMRI examinations: before MIST (which was directly after the emotional attention task), after each of the three MIST runs, as well as 10 and 20 min post MIST. All saliva samples were taken with approximately 10 min in-between. The Salivette collection tubes were kept at 4 °C, centrifuged at 1000× *g* for 1 min, transferred to Eppendorf tubes (Eppendorf, Germany), immediately frozen at −20 °C and subsequently stored at −80 °C until analysis. For biochemical analysis, samples were thawed and salivary cortisol levels determined using a chemiluminescence assay with high sensitivity and a minimal detection of 0.44 nmol/L (IBL, Hamburg, Germany) at Dresden Lab Service GmbH (Dresden, Germany). Intra- and inter-assay coefficients of variation were below 8%.

### 2.8. The Stroop Colour and Word Interference Test

A computerised version (Inquisit lab, Millisecond Inc., Seattle, WA, USA) of the Stroop colour and word interference test [24,25] was administered at the study visits before and after placebo and probiotic intervention, respectively. Pairs of conflicting stimuli (word and colour) were presented simultaneously on a computer screen, including the name of a colour printed in the ink of another colour. The difference between the response time when reading the words printed in conflicting colour and reading the words printed in the matching colour (same as the semantic meaning) was defined as the Stroop effect, which is a measure of cognitive flexibility and the ability to inhibit cognitive interference during a stressful condition [26]. 

### 2.9. Autonomic Nervous System Measurement

During the Stroop test before and after each of the intervention periods, autonomic nervous system activity was assessed, mainly based on heart rate measures, using the Biopac system and software (BioPac Inc., Goleta, CA, USA). This is a valid measure of stress, cognitive flexibility, neuropsychological abnormalities and psychopathology [27].

Electrocardiography (ECG) was continuously recorded at a sample rate of 1000 Hz using a Biopac MP150 system and a Biopac module (GSR100C) and transducer (TSD203) with electrodes placed in a bipolar precordial lead for ECG. Analysis was performed using Acknowledge 4.3.1 (BioPac Inc., Goleta, CA, USA). 

A custom-designed peak detection algorithm was used to determine the inter-beat intervals for assessment of average heart rate and heart rate variability (HRV). HRV is a peripheral response of ANS signalling. HRV measures included spectral analysis measures of the vagal (high frequency peak power) and sympathetic (low frequency peak power) responses (assessed based on RR intervals) and the ratio of these using the modified Pan-Tompkins algorithm of real time QRS-complex detection [28]. Artifacts were identified by visual inspection and modified using standard procedures, including smoothing of the signal, which does not affect the timing of the RR intervals. 

### 2.10. fMRI Analysis

Generally, nominal *p*-values are reported for *p* < 0.1 as well as Benjamini–Hochberg’s false discovery rate (FDR) estimates with a cutoff FDR < 0.05 and results after Bonferroni corrections before final conclusions.

#### 2.10.1. Preprocessing for fMRI Activity Analysis

The SPM12 (Statistical Parametric Mapping, The Wellcome Centre for Human Neuroimaging, UCL Queen Square Institute of Neurology, London, UK) default settings were used for data preprocessing if not specified otherwise (Matlab 9.3 R2017b, The Mathworks Inc., Natick, MA, USA). 

Functional images were co-registered with the structural high-resolution scan, normalised (warped) into Montreal Neurological Institute (MNI)-space and resampled to a voxel size of 3 × 3 × 3 mm^3^ before smoothing using a Gaussian kernel (smoothing width set to 6 mm). The series of ready-preprocessed fMRI NIfTI-files for each individual and visit was then used as input for the statistical analysis described below.

#### 2.10.2. fMRI Activity Analysis

Brain activity during the MIST was measured as a contrast of experimental and control conditions. The general linear model was used to model blood-oxygen-level-dependent (BOLD) time series as a function of explanatory variables or regressors.

First, we examined brain responses to the MIST paradigm after the placebo intervention in order to validate our setup of the paradigm and select regions of interest (ROIs) that overlapped with the a priori selected literature-based ROIs for the intervention contrast. Thereafter, we compared brain activity between the probiotic and placebo intervention. The interventions’ fMRI results were compared with respect to the differential activity evoked by the MIST paradigm for each of the three MIST runs separately.

#### 2.10.3. Model Specification

Statistical analyses were performed in R (version 4.0.0) [29], based on the expected BOLD response for the task indicator function given by the MIST design, as a convolution with the hemodynamic response function (HRF), proposed in the R-package “fmri” [30,31]. The linear model used for ANOVA analysis of fMRI activities, with regard to voxel, region and cluster levels respectively, was specified as follows:

(1) $$\mathrm{yi}=\mathrm{SUBJ}+\beta_{1}\times \mathrm{VOL}+\beta_{2}\times \mathrm{REST}+\beta_{3}\times \mathrm{PLUS}+\beta_{4}\times \mathrm{MINUS}+\varepsilon$$

where SUBJ denotes a subject-specific intercept, β_1_ denotes the coefficient of a linear drift effect (as slope for the covariate VOL, giving the number of measured brain volumes during the paradigm) for normalisation and β_2_ is a component in place for the resting parts of the paradigm (with REST as indicator variable). β_3_ denotes the coefficient of an average component, PLUS, of experimental and control parts of the paradigm, whereas β_4_ denotes the coefficient of a component specifying the differences between these two blocks (‘experimental’-‘control’) and thus reflects the pure stress response to the MIST paradigm. Both effects, PLUS and MINUS, are needed in the model to reflect all possible activations due to both parts of the paradigm, ‘experimental’ and ‘control’. ‘Experimental’ and ‘control’ were themselves specified as standard models of the BOLD HRF, with default parameters as proposed by Worsley et al. [31] and Polzehl and Tabelow [30].

This model was used to assess significant voxels/regions/clusters for which ‘experimental’-‘control’ was significantly different from zero with regards to brain activity after the placebo intervention. The model was analysed as a classical linear model (employing R-function lm), without taking any individual effects into account (no random effects modelled). Hence, results are expected to be conservative with regard to reporting significant voxel effects, which would be significant even on a pure group-level comparison. In contrast, for the analysis of intervention effects, individual effects (random effects) were modelled to enhance power, as detailed below.

#### 2.10.4. Implementation of the MIST Paradigm in Our Setting

The setup of the MIST paradigm was validated as described above to ensure that the same regions of altered brain activity as in an earlier publication [23] were found when comparing the control and experimental conditions based on brain activity after the placebo intervention.

#### 2.10.5. Region of Interest Approach

We used a stepwise process to define ROIs for the comparison of brain responses to the MIST paradigm for the probiotic intervention and the placebo intervention. First, a collection of a priori ROIs (*n* = 27) were selected based on relevant peer-reviewed research [21,23,32] (Supplementary Table S5). Second, the voxels/regions that showed a positive difference subtracting the BOLD signals of the control condition from the experimental condition (experimental > control) with regards to brain activity after the placebo intervention were used for modelling if differences were changing dependent on treatment. Finally, the overlap of regions that were involved in the task and among the literature-based selection were chosen as the final set of ROIs.

#### 2.10.6. Analysis of Intervention Effects

##### Region-Based Analysis

*p*-values of individual voxels’ linear models and their MINUS effects were assessed applying Bonferroni correction for level of significance and negative log10(*p*)-values saved as 3D-NIfTI files. For annotation of the regions, these *p*-value brain volumes in MNI space were displayed using a brainnetome atlas (BNA) [33]. Using an FSLview, the islands of significant *p*-values were examined visually and located within their functional BNA atlas regions. For analysis of the stress response of specific BNA regions, signals of all voxels of individual BNA regions were averaged. Those regions that overlapped with the collection of literature-based ROIs were selected for the region-based analysis (Supplementary Table S5). 

##### Cluster-Based Analysis

Additionally, cluster-based analysis was performed in order to focus on functionally altered areas instead of anatomically defined regions. Neighbouring voxels with altered signal intensities, as defined by a significant ‘experimental’-‘control’ effect in the voxelwise analysis (significant β_4_ in the model above, with nominal *p*-value < 10^−^^50^) were defined as clusters as detected by density-based spatial clustering [34,35] used with a critical epsilon neighbourhood set to eps = 1.5 and a minimum number of points set to minPts = 10. A number (*n* = 5 for run one, *n* = 4 for run two and *n* = 2 for run three) of those clusters was selected a priori (Supplementary Table S6). Clusters lining the skull/ outside the MNI template were not reported, since they were classified as potential artifacts. For each cluster, a peak coordinate was defined as the voxel with the most significant difference ‘experimental’-‘control’, i.e., showing a specific response to the stress-introducing part of the paradigm.

##### Statistical Analysis

For identifying intervention effects in the crossover design of the current study, a mixed effects model with fixed effects as in the model above (Equation (1)) plus a treatment effect and an interaction of treatment and ‘experimental’-‘control’ (MINUS) effect was built:

(2) $$\mathrm{yi}=\mathrm{SUBJ}+\beta_{1}\times \mathrm{VOL}+\beta_{2}\times \mathrm{REST}+\beta_{3}\times \mathrm{PLUS}+\beta_{4}\times \mathrm{MINUS}+\beta_{5}\times \mathrm{TREAT}+\beta_{6}\times \mathrm{TREAT}\times \mathrm{MINUS}+\varepsilon$$

In addition, random effects for the PLUS and MINUS effects, for the treatment effect (β_5_), and for the interaction of the ‘experimental’-‘control’ (MINUS) effect with the treatment effect (β_6_), were modelled to account for repeated data from the same individual. We report the test results for the fixed effect interaction of the ‘experimental’-‘control’ (MINUS) effect with the treatment effect, as this shows if the interventions with probiotics and placebo lead to differential effects regarding the MIST experimental versus control parts.

The models were analysed both at the levels of BNA regions as well as at the level of clusters as defined above. fMRI activity profiles for clusters and BNA regions were computed as averages of the composing voxels prior to modelling them as outlined. Accordingly, signal intensities of all composing voxels of individual sub-clusters were averaged for each brain volume separately, thus keeping the time course of the signal. 

#### 2.10.7. Task-Related fMRI Connectivity Analysis

The functional connectivity analysis was performed in CONN connectivity toolbox version 18.b standalone using Matlab 9.5 R2018b. The CONN default settings were used for data pre-processing. The CONN default pre-processing pipeline was used for data pre-processing with conservative settings (95th percentile) for ART-based outlier detection for scrubbing. Functional and structural scans were realigned, slice-time corrected, segmented and normalised into MNI-space before smoothing using a Gaussian kernel (smoothing width set to 8 mm). Denoising was performed with sequential regression (RegBP), a band-pass filter of 0.008 to 0.09 Hz, linear detrending and no despiking. The data of two subjects were excluded due to technical reasons. 

The interventions were compared with respect to the differential connectivity evoked by the challenge paradigm for each of the three MIST runs separately. A ROI-to-ROI analysis (bivariate correlations, HRF weighting) of clusters showing significant activation during the MIST after the placebo intervention was performed. As the initial cluster definition was based on the contrast between the experimental and the control condition, connectivity analysis could be performed for a comparison of the experimental conditions after both of the interventions. A two-sided, seed-level correction (FDR < 0.05) was used as threshold for the ROI-to-ROI connections for a matrix of 12 × 12 clusters in run one, 14 × 14 clusters in run two and 3 × 3 clusters in run three. 

#### 2.10.8. Visualisation of fMRI Data

The software multi-image analysis GUI (MANGO, Research Imaging Institute, University of Texas Health Science Center, San Antonio, TX, USA) was used to view functional images.

### 2.11. Analysis of Behavioural Data during MIST

In order to control if subjects engaged in the task, the number of tasks when participants pressed any button were assessed. In addition, the number of correct and incorrect responses as well as non-response trials within the time limit were assessed per condition and block.

### 2.12. Subjective Ratings

After the second fMRI visit, subjects were asked to rate their subjective stress perception during the fMRI itself and during the MIST at both fMRI visits on visual analogous scales (VAS, 0–100%). They were also asked to rate (using VAS) the extent to which they had suspected that the MIST had a purpose different from what they were told and the extent to which they had suspected that the MIST was meant to evoke stress. 

### 2.13. Statistical Analysis of Exploratory Outcome Parameters

Saliva samples collected during the fMRI experiments for measurement of salivary cortisol (six saliva samples at two occasions) were analysed using a repeated measures ANOVA of log-transformed baseline-corrected salivary cortisol concentrations with respect to treatment, time (sample order) and treatment-time interaction effects. Residuals for salivary cortisol analysis were checked for approximate normality by visual inspection (Supplementary Figure S3). Additionally, the area under the curve (AUC) and the total average of baseline-corrected salivary cortisol concentrations were assessed per subject. Intervention effects on these measures were analysed using a Wilcoxon matched-pairs signed rank test as data were not normally distributed (Shapiro-Wilk test) (GraphPad Prism 8, GraphPad Software Inc., San Diego, CA, USA). The results of one subject were excluded from salivary cortisol analysis as an outlier with the lowest value of this subject being more than 3.5 standard deviations higher than the average value of all values from all other subjects.

The data of the Stroop test, ANS activity, subjective stress ratings and behavioural data from the MIST task were tested for normal distributions using the Shapiro–Wilk test. The baseline-corrected effects of probiotics and placebo, respectively, were analysed using a Wilcoxon matched-pairs signed rank test for non-normally distributed data (Stroop, subjective stress ratings, behavioural data from MIST) and using a paired t-test for normally distributed data (HRV, sympathetic and vagal activity) (GraphPad Prism 8 and SPSS Statistics version 26, IBM Corp., North Castle, NY, USA).

Results are presented as medians and interquartile ranges (IQR 25 to 75).

## 3. Results

### 3.1. Subject Characteristics

Twenty-two healthy participants (16 women, 6 men) with a mean age of 24.2 ± 3.4 participated in the study. Their baseline characteristics are reported in Supplementary Table S7. Adverse events reporting is found in Supplementary Table S8.

### 3.2. Subjects Engaged in the MIST

There was no statistical difference in terms of any of the assessed engagement measures between the three MIST runs or the interventions, indicating that subjects were equally engaged throughout the task (Supplementary Table S9). The variation in total tasks during the experimental condition was in general very small and in all three MIST runs, subjects answered more tasks correctly in the control condition than in the experimental condition. This indicated that the difficulty level was manipulated well to be just beyond the subjects’ cognitive performance during the experimental condition. 

### 3.3. The MIST Paradigm Worked Well in Our Setting (Paradigm Validation)

In line with previous findings [23], significantly increased activation (experimental > control) was observed in several regions implicated in stress response and emotional regulation, including the occipital cortex, prefrontal cortex, cingulate cortex, and limbic regions (BNA region-based analysis; Bonferroni-corrected; *p* < 0.05/246). These regions were used for subsequent analyses of changes in task-related activity in comparing the probiotic and placebo interventions (Supplementary Table S5).

### 3.4. Probiotics Differentially Affected Brain Activity during MIST

In order to investigate the effects of the probiotic intervention on brain function during the different phases of stress, brain activity during the three MIST runs was analysed separately.

#### 3.4.1. Region-Based Analysis

Region-based analysis indicated a subtle difference in brain activity for experimental versus control condition when comparing the interventions, indicating a slight difference in brain activity during the MIST. 

Changes in activation (*p* < 0.1) were observed in several regions that were among the predefined ROIs (without correction for multiplicity): Most regions showed a reduced activation (probiotics < placebo), including regions in the orbital and cingulate gyri. Only four of the significant BNA regions showed increased activity (probiotics > placebo), none of which was among the predefined ROIs. Several of those regions with altered brain activity are implicated in emotion, stress and cognition. The results separated by the three MIST runs are reported in Table 1. 

#### 3.4.2. Cluster-Based Analysis

In addition to analysis of anatomically defined BNA regions, cluster-based analysis was performed in order to also focus on functionally altered areas. Clusters that showed significant changes in activation when comparing the experimental with the control condition after placebo intervention and were of interest based on previous literature were selected for analysis. 

**Figure 2:**
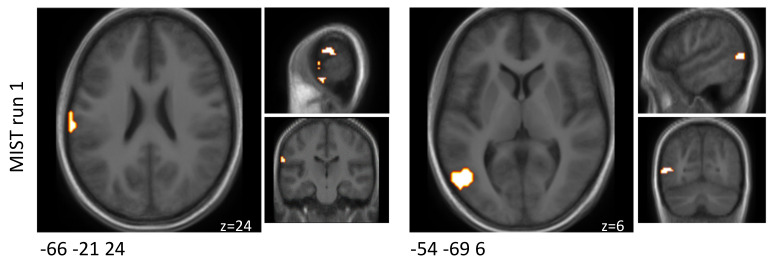
Schematic visualisation of clusters that were found to be associated with changes in brain activity (*p* < 0.1) when comparing the probiotic and the placebo interventions (probiotic > placebo) and when comparing the experimental and control condition before multiplicity correction during the MIST paradigm. Clusters are superimposed on average anatomical scans. Clusters can be identified by the coordinates of their peak (x y z).

Using cluster-based analysis, activation (probiotics > placebo) (*p* < 0.1, without correction for multiplicity) was observed in two clusters when comparing the interventions with respect to the differential activity evoked by the challenge paradigm in MIST run one. Those two clusters were located in the rostroventral and dorsolateral areas. In MIST run two and three, no significant differences were found by cluster-based analysis. The results separated by the three MIST runs are reported in Table 2 and Figure 2.

### 3.5. Probiotics Significantly Affected Functional Brain Connectivity during MIST

In order to understand how the probiotic intervention altered task-related functional connectivity, a connectivity analysis was performed between all clusters that were found to be differentially activated when comparing the experimental and the control condition after the placebo intervention.

**Figure 3:**
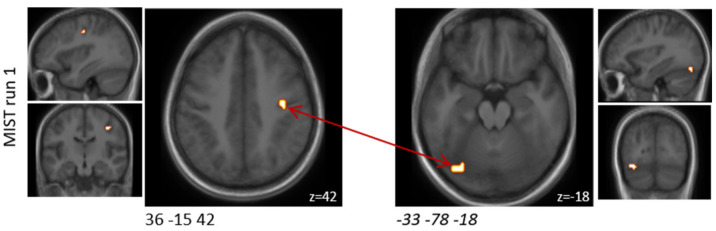
Schematic visualisation of clusters that were found to be associated with significant (FDR-corrected *p* < 0.05) functional connectivity changes when comparing the probiotic and the placebo interventions (probiotic > placebo) during the MIST paradigm. One of these clusters covered a predefined ROI (italic). Clusters are superimposed on average anatomical scans. Clusters can be identified by the coordinates of their peak (x y z).

In MIST run one, functional connectivity between two of these clusters was found to be significantly (FDR-corrected *p* = 0.02) increased by the probiotic intervention (Table 3 and Figure 3). These two clusters were located in the upper limbic and medioventral regions; the latter was among the predefined ROIs. During the MIST runs two and three, no alterations in functional connectivity were observed.

### 3.6. Stress Induction during MIST Was Not Significantly Affected by Probiotics Intake

**Figure 4:**
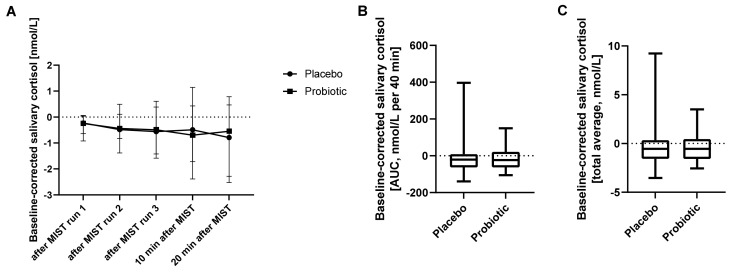
Salivary cortisol concentration during MIST. (**A**) Baseline-corrected salivary cortisol concentration during and after MIST. ANOVA, *n* = 21, median with interquartile range. (**B**) Salivary cortisol as area under the curve (AUC) and (**C**) as total average. Line presents median, box presents 25th and 75th percentile, and whiskers present minimum to maximum. Wilcoxon matched-pairs signed rank test, *n* = 21.

Stress-inducing effects of the fMRI MIST paradigm were assessed by salivary cortisol (assessed after each MIST run) and self-rated questionnaires (comparing responses for the two intervention periods). A repeated-measures ANOVA of salivary cortisol levels during the fMRI experiments revealed no significant differences between the two treatments (*p* = 0.99), nor did they show a significant change over time (*p* = 0.59) or a treatment-time interaction effect (*p* = 0.72) (Figure 4). An analysis of baseline-corrected salivary cortisol AUC (*p* = 0.71) and total average (*p* = 0.95) by Wilcoxon matched-pairs signed rank test revealed no intervention effect either.

Subjects felt more stressed by the MIST than by the fMRI examination itself based on subjective stress ratings on a VAS. Median stress ratings for the fMRI examination itself were 32.5% (IQR 7.6 to 54.3) at the first occasion and 17.3% (IQR 6.0 to 39.6) at the second occasion. Subjects felt significantly less stressed by the MIST at their second fMRI examination, independent of the intervention (median at first fMRI: 70.8% (IQR 57.6 to 90.0); median at second fMRI: 61.8% (IQR 37.9 to 70.8), *p* = 0.001). The intervention had no effect on subjects’ subjective stress perception during the MIST (*p* = 0.76) nor on their stress perception by the fMRI investigation itself (*p* = 0.43). Participants highly suspected that the MIST was meant to measure something different than they were told with a median rating of 79.3% (IQR 50.9 to 93.1) and with a median rating of 89.8% (IQR 63.5 to 99.6) that it was meant to provoke stress. 

### 3.7. Cognitive Performance Was Not Significantly Affected by the Probiotic Intervention

**Figure 5:**
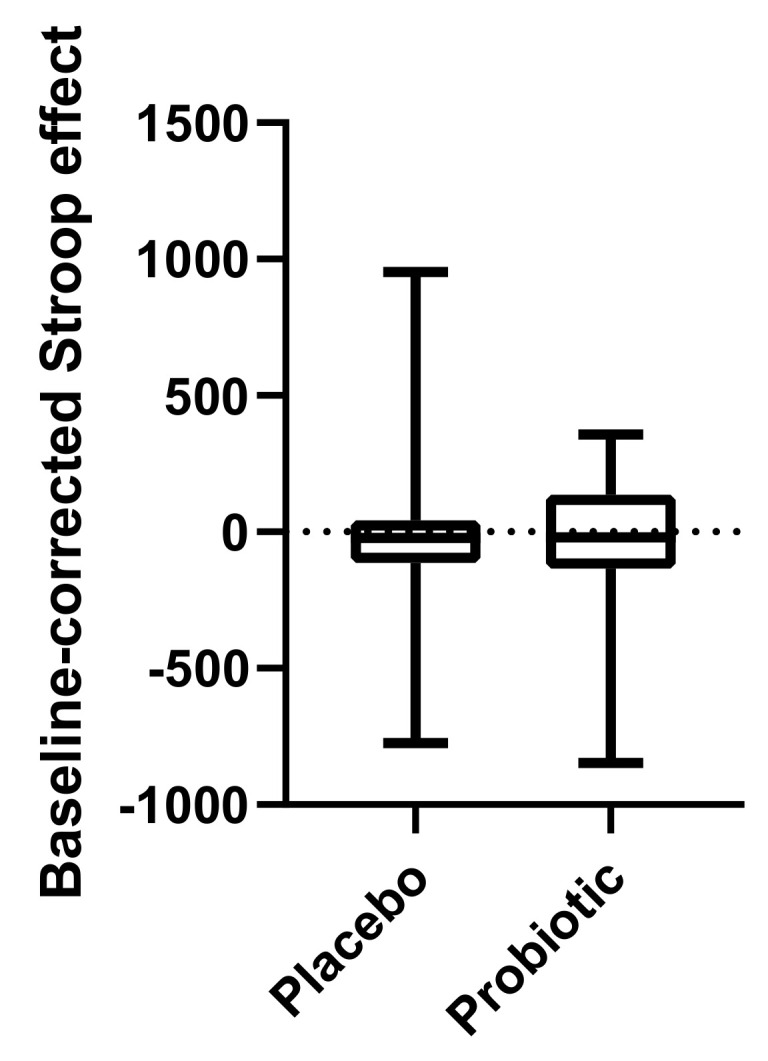
Cognitive performance measured as Stroop effect (baseline-corrected, after-before intervention). Line presents median, box presents 25th and 75th percentile, and whiskers present minimum to maximum. Wilcoxon matched-pairs signed rank test, *n* = 22.

A Wilcoxon matched-pairs signed rank test revealed that the Stroop effect, a measure of cognitive performance during acute stress, was not significantly differentially affected by the probiotics compared to the placebo (*p* = 0.59) (Figure 5).

### 3.8. Autonomic Nervous System Activity Was Not Significantly Affected by the Probiotic Intervention

**Figure 6:**
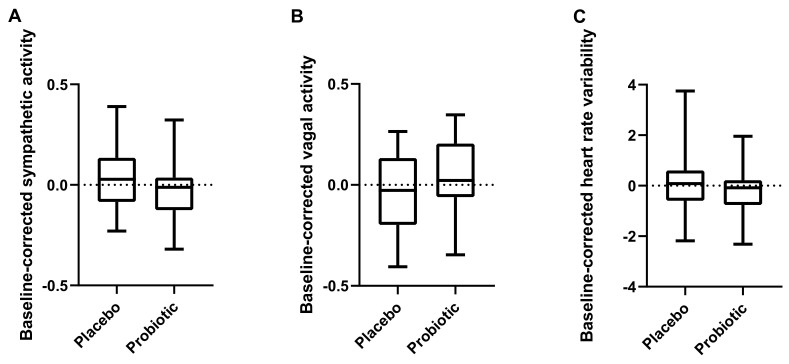
Autonomic nervous system activity during stress (evoked by the Stroop task). (**A**) sympathetic activity, (**B**) vagal activity, and (**C**) heart rate variability. Line presents median, box presents 25th and 75th percentile, and whiskers present minimum to maximum. Paired *t*-test, *n* = 22.

Baseline-corrected sympathetic (*p* = 0.19) and vagal activity (*p* = 0.21) during a stressful situation (evoked by the Stroop test) was not significantly affected by probiotics intake. Similarly, baseline-corrected HRV was not significantly affected (*p* = 0.51) (Figure 6).

## 4. Discussion

Here, we present the first study to use the MIST paradigm in a probiotic intervention study. Overall, this is one of the first studies to demonstrate that probiotic supplementation affects brain activity during acute psychological situations, such as stress. We found that the probiotic intervention altered activity in specific brain regions known to regulate emotion and stress response, including regions of the lateral orbital and ventral cingulate gyri, although these differences did not withstand correction for multiple comparisons. Furthermore, we found that the probiotic intervention significantly altered functional connectivity between the upper limbic and medioventral regions. Interestingly, we found that the probiotic intervention did not affect the activation of other limbic or subcortical areas known to be activated by the MIST paradigm, including the hippocampus, amygdala, hypothalamus, and striatum [23].

We also present one of the first studies to investigate ANS function during acute psychological stress in a probiotic intervention study. We found that cognitive performance and ANS function during the Stroop task were not significantly affected by the probiotic intervention.

We were able to show that the MIST paradigm worked well in our fMRI setting by observing altered brain activity in response to the paradigm independent of intervention, which was comparable with previous findings [23]. Furthermore, subjects were engaged in the task and subjectively rated the task as stressful.

Thereafter, intervention effects were investigated. The selection of ROIs was based on expectations of intervention effects and mostly focused on regions that are implicated in emotion, perception and stress. A number of brain regions affected by the intervention are also involved in cognition. By adopting an exploratory analysis approach, we were able to also identify effects of the probiotic intervention on these brain regions that were expected to be involved in the cognitive part of the task, but were not among our ROIs.

In the present study, we decided to analyse the three MIST runs separately to increase the temporal resolution. This allowed us to analyse the effects of the probiotic intervention on brain activity in the presence of repeated stress exposure, including investigating potential mental fatigue and adaptive stress responses. We chose this procedure since EEG measures have shown an association between fatigue and altered activation in the cingulate cortex [36], a brain region whose resting state EEG was shown to be altered after a four-week intervention with *Bifidobacterium longum* in healthy subjects [18]. In our study, the same region showed deactivation in MIST run two upon probiotic intervention. These findings suggest the novel possibility that probiotic interventions might have fatigue effects.

Previously reported studies typically have analysed all MIST runs combined at a cost of a lower temporal resolution. However, the brain activity and connectivity results of the present study differed between the three MIST runs. The probiotic intervention seemed to have predominantly affected the first MIST run both in terms of brain activity and functional connectivity alterations. Although not much overlap could be observed between the runs, a few brain regions showed similar response patterns to the arithmetic stress paradigm between two runs. Regions of the orbital gyrus and the insular gyrus showed decreased activation after probiotic intervention in both runs one and two. One area of the precentral gyrus also showed decreased activation with probiotic intervention in all three runs. 

The different effects of the probiotic intervention on brain function during the three MIST runs may be explained by the different phases of stress responses [21,22]. Activation of the HPA axis starts with the release of corticotropin-releasing hormone (CRH) from the hypothalamus, which in turn stimulates the secretion of adrenocorticotropic hormone (ACTH) from the anterior pituitary. ACTH acts on the adrenal cortex, which is responsible for glucocorticoid production (mainly cortisol). Finally, glucocorticoids act back on the hypothalamus and pituitary to suppress CRH and ACTH production in a negative feedback cycle [1]. During the first MIST run, stress is initialised; thus, the brain activity and connectivity are investigated during stress, but without glucocorticoid signalling [21,22]. As the MIST paradigm proceeds, glucocorticoids (mainly cortisol) are produced in the periphery and signalled back to the brain. Thus, during the second MIST run, brain activity and connectivity are investigated under stress in the presence of some glucocorticoids. During MIST run three, additional amounts of glucocorticoids are present. In the present study, most differences upon probiotic intervention were seen in the first MIST run, whereas fewer differences were seen in the later MIST runs, which are known to be associated with increases in cortisol signalling. The probiotic intervention did not affect salivary cortisol concentration during MIST, further supporting a role for the probiotic intervention in modulating stress response prior to glucocorticoid secretion. This implies that the probiotic intervention primarily impacts initial stress responses in the absence of glucocorticoid signalling. Similarly, salivary cortisol during another stress paradigm (socially evaluated cold pressure test) was reported not to be affected by probiotic intervention [17]. In addition, cortisol levels as a marker of baseline stress were reported to be only marginally affected, if at all, by probiotic supplementation [37,38,39]. One study reported probiotic effects on salivary cortisol; however, several different statistical analysis methods were applied [15].

The present study benefited from its crossover design with each participant serving as their own control. Nevertheless, as order effects were not taken into account for analysis, intervention effects may be biased. Due to its proof-of-concept character, the sample size of the present study was relatively small and was not sufficient for subgroup analysis. Subgroup analysis would be specifically interesting in terms of responders versus non-responders to stress induction by the MIST paradigm as reported previously [23]. Additionally, for other types of acute psychological stress tasks, increased subjective stress ratings without concomitant increase in cortisol levels have been reported [40]. Results from subjects who did experience the task as stressful, but who did not show an increase in salivary cortisol, may have masked the effects that would be observed if only focusing on those subjects who showed an increased stress response. However, the crossover design of the present study may have weakened the impact of such subgroups, as one could speculate that subjects would respond in similar patterns to the task after both interventions, as the overall cortisol response did not differ between interventions. Nevertheless, future studies with a larger sample size should consider responder versus non-responder analyses. Notably, the presented study assessed men and women without any subgroup analysis for the above reasons, although stress responses between men and women may differ. However, the employed crossover design attenuates a potential sex bias. It should also be noted that the probiotic mixture contained additional bioactive compounds that could affect the central nervous system; however, their impact should be negligible in the present setting of a well-nourished study population, since levels were within typical daily intake measures.

The subtle effects observed in the present proof-of-concept study are especially important in the light of a rather mild non-pharmaceutical intervention in a healthy study population. Effects in a diseased population can be expected to be even stronger, although hard to identify due to a heterogenous population with relatively high intra-individual variations [41]. Interestingly, such subtle effects could be seen already after the rather short intervention period of four weeks. Even shorter intervention periods have evoked probiotic effects, e.g., on the small intestinal level [42]. This indicates that the probiotic effects in this study could also be initiated in the small bowel, the common habitat for especially *Lactobacilli*, which was contained in the studied probiotic mixture. Long-term probiotic interventions could thus be regarded to have clear non-pharmaceutical therapeutic potential.

## 5. Conclusions

The present study suggests that the investigated probiotic mixture of *Lactobacillus helveticus* R0052, *Lactiplantibacillus plantarum* R1012 and *Bifidobacterium longum* R0175 could subtly dampen brain responses during the MIST, which reflects an acute stress situation. Since probiotics generally may maintain the health of healthy subjects as well as improve the symptoms of patients, this study could further lead to possible clinical implications for improving stress resilience and potential roles in the treatment of affective and gut-brain axis disorders.

## Figures and Tables

**Table 1:** BNA regions that were found to be associated with changes in brain activity (*p* < 0.1) between both interventions when comparing the experimental and control condition before multiplicity correction during the MIST paradigm. Several of these BNA regions were among the predefined ROIs (italics).

BNA Region	Experimental-Control in Probiotic-Placebo *p*-Value *	Experimental-Control in Probiotic-Placebo Effect Size	Anatomical Region
MIST run 1			
62	0.022	−1.722	Precentral gyrus, A4tl, area 4 (tongue and larynx region)
*46*	*0.036*	*−2.359*	*Orbital gyrus, A11l, lateral area 11*
168	0.064	−2.043	Insular gyrus, dla, dorsal angular insula
174	0.084	−1.689	Insular gyrus, dld, dorsal dysgranular insula
MIST run 2			
62	0.009	−1.805	Precentral gyrus, A4tl, area 4 (tongue and larynx region)
81	0.015	1.223	Middle temporal gyrus, A21c, caudal area 21
40	0.028	−1.586	Inferior frontal gyrus, A44v, ventral area 44
174	0.033	−1.298	Insular gyrus, dld, dorsal dysgranular insula
142	0.035	−1.332	Inferior parietal lobule, A40c, caudal area 40 (PFm)
92	0.038	−3.248	Inferior temporal gyrus, A37elv, extreme lateroventral area 37
*182*	*0.057*	*−1.590*	*Cingulate gyrus, A23v, ventral area 23*
38	0.067	−1.379	Inferior frontal gyrus, A44op, opercular area 44
*46*	*0.068*	*−2.077*	*Orbital gyrus, A11l, lateral area 11*
*181*	*0.075*	*−1.858*	*Cingulate gyrus, A23v, ventral area 23*
MIST run 3			
62	0.027	−0.967	Precentral gyrus, A4tl, area 4 (tongue and larynx region)
39	0.028	−1.442	Inferior frontal gyrus, A44v, ventral area 44
193	0.085	1.400	Medio ventral occipital cortex, cCunG, caudal cuneus gyrus
97	0.087	2.300	Inferior temporal gyrus, A37vl, ventrolateral area 37
112	0.096	0.866	Parahippocampal gyrus, A35/36c, caudal area 35/36

* After correction for multiple testing using Bonferroni, none of these alterations passed the level of significance (*p* > 0.05/246). BNA—Brainnetome atlas, MIST—Montreal Imaging Stress Task, ROI—Region of interest.

**Table 2:** Clusters that were found to be associated with changes in brain activity (*p* < 0.1) between both interventions when comparing the experimental and control condition before multiplicity correction during the MIST paradigm.

MNI Coordinates of Peak (x y z)	Cluster Size [mm^3^]	Experimental-Control in Probiotic-Placebo *p*-Value *	Experimental-Control in Probiotic-Placebo Effect Size	Anatomical Region
MIST run 1				
−66 −21 24	1026	0.070	1.462	A40rv, rostroventral area 40 (PFop)
−54 −69 6	1350	0.077	2.871	A37dl, dorsolateral area 37
MIST run 2				
none				
MIST run 3				
none				

* After correction for multiple testing using Bonferroni, none of these alterations passed the level of significance (MIST run 1: *p* > 0.05/12; MIST run 1: *p* > 0.05/14; MIST run 3: *p* > 0.05/3). MIST—Montreal Imaging Stress Task, MNI—Montreal Neurological Institute.

**Table 3:** Clusters that were found to be associated with significant functional connectivity changes between both interventions during the MIST paradigm. One of these clusters covered a predefined ROI (italic).

MNI Coordinates of Peak (x y z)	Cluster Size [mm^3^]	Anatomical Region	MNI Coordinates of Peak (x y z)	Cluster Size [mm^3^]	Anatomical Region	T Probiotic-Placebo	FDR Probiotic-Placebo
MIST run 1							
36 −15 42	297	A4ul, area 4 (upper limb region)	*−33 −78 −18*	*405*	*A37mv, medioventral area37*	3.6	0.021
MIST run 2							
none							
MIST run 3							
none							

FDR—False discovery rate, MIST—Montreal Imaging Stress Task, MNI—Montreal Neurological Institute, ROI—Region of interest.

## Data Availability

Part of the underlying data can be found in the Supplementary Materials. Regarding the fMRI data: underlying deidentified data will be made available upon request pending ethical approval and a signed agreement. Scripts of the performed analysis will be made available upon request.

## Supplementary Materials

The following are available online at https://www.mdpi.com/article/10.3390/nu14071329/s1, Sample size justification, Figure S1: Participant flow chart; Figure S2: Scheme of the Montreal Imaging Stress Task (MIST); Figure S3: Residual plot for salivary cortisol concentration; Table S1: Exclusion criteria, Table S2: Composition of the probiotic product; Table S3: Compliance by intervention period; Table S4: Compliance by product; Table S5: Regions of interest; Table S6: Clusters; Table S7: Baseline characteristics; Table S8: Adverse events; Table S9: Behavioural data during MIST.
