# Supplementary material for: Probiotic Mixture Containing Lactobacillus helveticus, Bifidobacterium longum and Lactiplantibacillus plantarum Affects Brain Responses to an Arithmetic Stress Task in Healthy Subjects: A Randomised Clinical Trial and Proof-of-Concept Study
Source: Nutrients. 2022 Mar 22;14(7):1329. doi: 10.3390/nu14071329 (PMC9002567; doi:10.3390/nu14071329)
Supplement: Supplementary file 1 [file nutrients-14-01329-s001.zip › Probio_ManusMIST_Supplement.pdf]

SUPPLEMENTARY INFORMATION

Probiotic mixture containing *Lactobacillus helveticus*, *Bifidobacterium longum* and *Lactiplantibacillus plantarum* affects brain responses to an arithmetic stress task in healthy subjects: A randomised clinical trial and proof-of-concept study

Hanna MT Edebol Carlman[*] and Julia Rode[*], Julia König, Dirk Repsilber, Ashley N Hutchinson, Per Thunberg, Jonas Persson, Andrey Kiselev, Jens C Pruessner, Robert J Brummer.

* Equal contribution/ shared first authorship

Sample size

Sample size calculation was performed considering the primary outcome, alternations in brain activity and functional connectivity, taking care of the crossover design in our study, as the comparison of the two interventions (probiotics versus placebo) within the same subject. Calculations were based on a paired t-test to indicate a change in connectivity as large as the observed standard deviation for a single functional connection (measured as correlation) between two given brain regions, i.e. aiming to be able to detect an effect size of $Cohen's\ dz = 1$. We aimed at a power of 80%, a 95% confidence interval, and Bonferroni correction for the multiple brain regions analysed (5 defined brain regions yield 10 interactions). Considering these constraints, a minimum of 18 subjects would be required to demonstrate a mean difference of f=u2-u1=0.3 (sd=0.3, Cohen's dz=1), i.e. for example a difference between partial correlations u1=0.3 and u2=0.6, at α=0.05 and for a maximum dropout rate of 20%.

Supplementary Table S1. Exclusion criteria

1. Concurrent or recent (< twelve weeks) treatment with drugs affecting intestinal function or mood, e.g. antidepressants or antibiotics
2. Concurrent or recent (< four weeks) use of nutritional supplements or herb products affecting intestinal function or mood (e.g. aloe vera, St. John's Wort, fibres, prebiotics and probiotics)
3. Diagnosis of major psychiatric or somatic disease
4. Abuse of alcohol or drugs
5. Recent (< four weeks) intake of proton pump inhibitors (e.g. omeprazol)
6. Asthma
7. Cardiovascular diseases
8. Epilepsy
9. Renal failure
10. Cerebral bleeding or history of cerebral bleeding
11. Allergy to latex
12. Pregnancy (assessed by urine test) or breastfeeding
13. Claustrophobia
14. Smoking or using tobacco including snuff
15. Inability to maintain exercise routine and dietary pattern during the study
16. Consumption of more than six cups of coffee/caffeine-containing beverages per day
17. Professional athlete
18. Any contraindication to an MRI (e.g. medical implant or device not compliant with MRI)
19. Recent (< three months) regular intake of systemic corticosteroids and anti-inflammatory medication (including non-steroidal anti-inflammatory drugs)
20. Known allergy to milk or soy
21. Any other reason the investigator felt the subject was not suitable for participation in the study

MRI – Magnetic resonance imaging

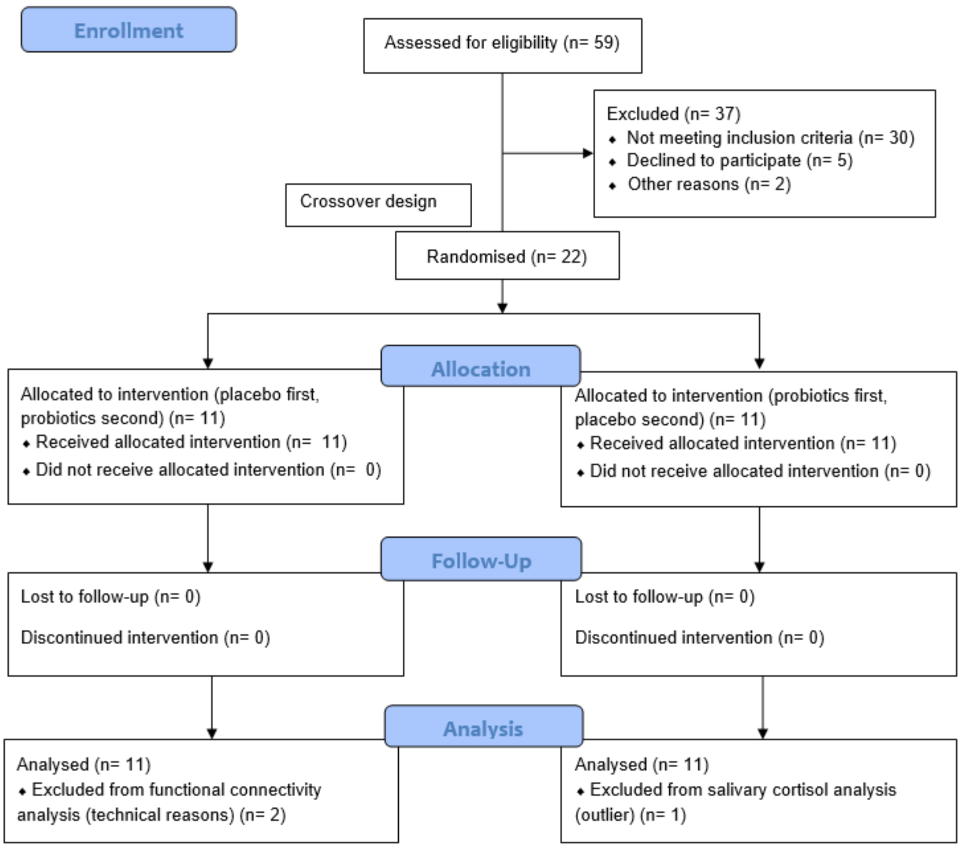

Supplementary Figure S1. Participant flow chart Supplementary

Supplementary Table S2. Composition of the probiotic product

**Active Ingredients**	**Amount per sachet**
**Inulin**	500 mg
**Magnesium**	45 mg
**Potassium**	50 mg
**Zinc**	9 mg
**Glutathione**	20 mg
**Lactoferrin**	10 mg
**Probiotic blend:** ***L. helveticus R0052 (CNCM-I-1722), B. longum R0175 (CNCM-I-3470), L. plantarum R1012 (CNCM-I-3736)***	3 x 10^9 CFU

Ingredients:
Fructose, Inulin, Magnesium Gluconate; Acidifying agent: Citric Acid; Aroma, Potassium Citrate, Mixture of live lactic cultures (*Lactobacillus Acidophilus* (*Helveticus*), *Bifidobacterium Longum*, *Lactiplantibacillus Plantarum*), Zinc Gluconate, Magnesium Oxide; Anti-caking agent: Silicon Dioxide; Glutathione; Sweeteners: Acesulfame K, Sucralose; Maltodextrin, Lactoferrin; Dyes: E102, E124.
(underlined ingredients were also contained in the placebo)

Supplementary Table S3. Compliance by intervention period All subjects consumed 90% or more of the study product.

	**Daily consumption of the study product**	**One single day consumption missed during second intervention**	**One day consumption missed in both interventions**	**Several days consumption missed during first intervention and one day missed during second intervention**
**Number of subjects**	14 (63.6%)	4 (18.2%)	2 (9.1%)	2 (9.1%)*

*Two subjects forgot to consume the intervention product during 3 and 4 days, respectively, during the first intervention period with a maximum of two missing consecutive days.

Supplementary Table S4. Compliance by product
All subjects consumed 90% or more of the study product.

	Daily consumption of the study product	One single day consumption missed	Several days consumption missed
**Placebo**	14 (63.6%)	8 (36.4%)	0 (0.0%)
**Probiotics**	18 (81.8%)	2 (9.1%)	2 (9.1%)*

*Two subjects forgot to consume the intervention product on 3 and 4 days, respectively. No more than two of those happened on consecutive days.

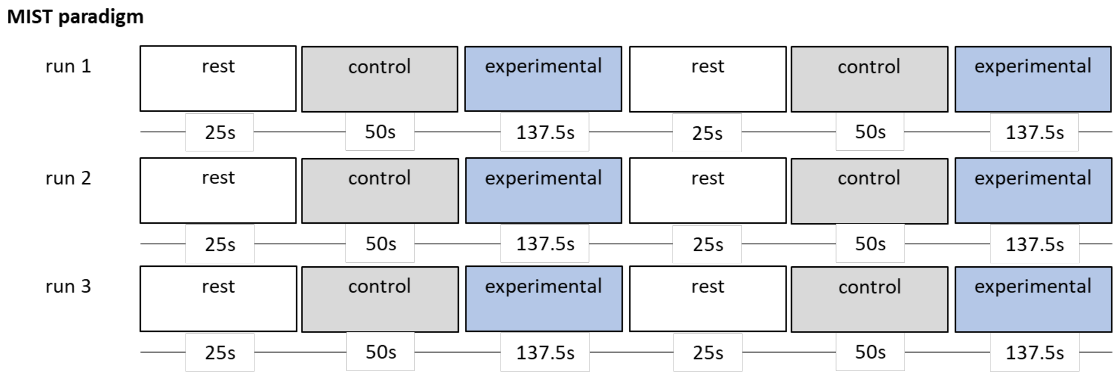

Supplementary Figure S2. Scheme of the Montreal Imaging Stress Task (MIST)

Supplementary Table S5. Regions of interest
All regions of interest that have been selected based on previous literature are presented. Regions that were significantly activated during the task (experimental>control) after the placebo intervention are marked in italics. Multiple comparison correction with Bonferroni. Significance level $p<0.05/246$.

BNA region	Anatomical region
211, 212	Medial amygdala
213, 214	Lateral amygdala
237, 238	*Rostral temporal thalamus*
215, 216	Rostral hippocampus
217, 218	Caudal hippocampus
165, *166*	*Insular cortex, ventral agranular insular*
169, *170*	*Insular cortex, ventral granular insular*
223, 224	*Nucleus accumbens*
219, 220	*Ventral caudate*
225, 226	*Ventromedial putamen*
175, 176	Cingulate cortex, dorsal area 23
177, 178	*Cingulate cortex, rostroventral area 24*
179, 180	*Cingulate cortex, pregenual area 32*
181, 182	*Cingulate cortex, ventral area 23*
185, 186	Cingulate cortex, caudal area 23
187, 188	*Cingulate cortex, subgenual area 32*
69, 70	*Superior temporal gyrus, medial area 38*
79, 80	*Superior temporal gyrus, rostral area 22*
41, 42	*Orbital gyrus, medial area 14*
49, 50	Orbital gyrus, area 13
45, 46	*Orbital gyrus, lateral area 11*
51, *52*	*Orbital gyrus, lateral area 12/47*
7, 8	Superior frontal gyrus, dorsolateral area 6
11, 12	*Medial prefrontal cortex, medial area 9*
13, 14	Medial prefrontal cortex, medial area 10
5, 6	Dorsolateral prefrontal cortex, lateral area 9
19, 20	Dorsolateral prefrontal cortex, area 46

BNA – Brainnetome atlas

Supplementary Table S6. Clusters

Clusters that were activated by the task (experimental>control) after the placebo intervention are presented. The clusters that cover the selected regions of interest defined by previous literature and data-driven are marked in italics. Multiple comparison correction with Bonferroni.

MNI coordinates of peak (x y z)	Cluster size [mm^3]	Anatomical region
**MIST run 1**		
63 12 6	972	Area 4 (tongue and larynx region)
45 -66 9	*3321*	*Area V5/MT+*
36 -15 42	297	Area 4 (upper limb region)
24 -81 27	*648*	*Medial superior occipital gyrus*
15 -33 45	702	Area 1/2/3 (lower limb region)
6 30 -30	*1566*	*Medial area 11*
0 0 39	*1215*	*Caudodorsal area 24*
-12 -69 36	270	Dorsomedial parietoccipital sulcus (Per)
-33 -78 -18	*405*	*Medioventral area37*
-54 -69 6	1350	Dorsolateral area37
-66 -21 24	1026	Rostroventral area 40 (PFop)
-66 -36 30	513	Caudal area 40 (PFm)
**MIST run 2**		
60 -39 21	1377	Caudoposterior superior temporal sulcus
42 -66 9	13095	Caudal area 39 (PGp)
51 15 0	810	Ventral area 44
9 -45 21	2322	Ventral area 23
27 -33 9	540	Posterior parietal thalamus
18 -24 15	486	Dorsal caudate
3 33 -33	*675*	*Area 13*
6 -48 51	459	Medial area 5 (Pem)
-9 -66 33	351	Dorsomedial parietoccipital sulcus (Per)
-9 -48 27	*270*	*Dorsal area 23*
-18 -24 21	648	Dorsal caudate
-27 -69 0	*1026*	*Medioventral area37*
-42 -69 12	*3159*	*Area V5/MT+*
-57 -57 -33	135	Cerebellum
**MIST run 3**		
3 33 -33	*135*	*Area 13*
-39 -81 -36	216	Cerebellum
-57 -36 -12	*648*	*A20cl, caudolateral area or 20*

MIST – Montreal Imaging Stress Task; MNI – Montreal Neurological Institute

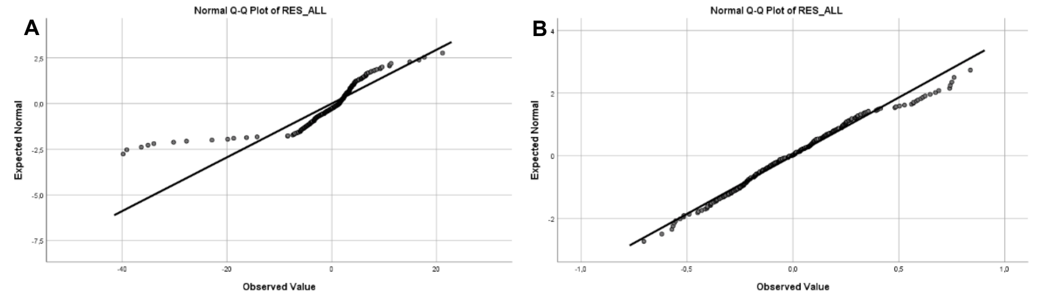

Supplementary Figure S3. Residual plot for (A) salivary cortisol concentration on original scale and (B) on logarithmic scale.

Supplementary Table S7. Baseline characteristics

**Age**	24.2 ± 3.4 years
**Male/female**	6/16
**BMI***	22.3 kg/m^2 (21.2 to 23.8)
**Hospital Anxiety and Depression Scale***	
• **Total score**	5.0 (4.0 to 8.0)
• **Depression subscore**	1.0 (0.8 to 2.0)
• **Anxiety subscore**	4.0 (2.0 to 6.3)
**State and Trait Anxiety Inventory***	
• **State subscore**	26.0 (22.0 to 30.5)
• **Trait subscore**	28.0 (25.0 to 33.3)
**Perceived Stress Scale (total score)***	9.0 (5.8 to 12.3)
**Quality of life (based on EQ-5D-5L index value)***	80.5% (75.0 to 90.0)

*For BMI and all questionnaire scores median and interquartile range (IQR 25 to 75) are reported
BMI – Body mass index

Supplementary Table S8. Adverse events
The total number of adverse events is reported, the number of those events which were suspected to be related to the intervention or possible related to the intervention are shown in brackets: total number (suspected/possible).

Symptom	Placebo	4 weeks after placebo	Probiotic	4 weeks after probiotic
**Abdominal pain**	2 (0/2)	0 (0/0)	1 (0/1)	0 (0/0)
**Diarrhoea**	0 (0/0)	1 (0/0)	2 (0/2)	0 (0/0)
**Bloating**	1 (0/1)	0 (0/0)	1 (0/1)	0 (0/0)
**Nausea**	1 (0/1)	0 (0/0)	2 (0/2)	1 (0/0)
**Headache**	12 (4/1)	1 (0/0)	5 (0/2)	0 (0/0)
**Cold**	14 (0/0)	0 (0/0)	6 (0/1)	3 (0/0)
**Sore throat**	1 (0/0)	0 (0/0)	0 (0/0)	1 (0/0)
**Fever**	1 (0/0)	0 (0/0)	1 (0/0)	0 (0/0)
**Sleep problems**	2 (0/2)	0 (0/0)	1 (0/1)	0 (0/0)
**Depressive symptoms**	0 (0/0)	1 (0/1)	0 (0/0)	1 (0/1)
**Anxiety symptoms**	0 (0/0)	0 (0/0)	1 (0/1)	0 (0/0)
**Pain in the knee**	0 (0/0)	0 (0/0)	2 (0/0)	0 (0/0)
**Fungal infection**	1 (0/1)	0 (0/0)	0 (0/0)	0 (0/0)
**Pollen allergy**	1 (0/0)	0 (0/0)	4 (0/0)	0 (0/0)

Supplementary Table S9. Behavioural data during MIST

	**Control: incorrect answers**	**Control: correct answers**	**Experimental: incorrect answers**	**Experimental: correct answers**	**Experimental: timeout**	**Experimental: timeout without pressing any button***	**Experimental: total number of tasks**
**MIST run 1**							
**Placebo**	1.0 (0.0-2.3)	11.5 (8.0-15.5)	8.5 (3.0-11.3)	30.0 (26.0-33.0)	23.5 (17.3-29.3)	4.5 (3.0-7.3)	60.5 (60.0-61.3)
**Probiotic**	1.0 (1.0-2.0)	13.5 (10.8-16.0)	7.0 (3.8-10.0)	29.5 (26.0-32.0)	25.0 (21.5-27.0)	5.0 (2.8-7.3)	61.0 (59.0-61.3)
**MIST run 2**							
**Placebo**	1.0 (1.0-3.0)	14.0 (11.8-16.3)	6.0 (3.8-7.3)	31.0 (28.8-33.3)	24.0 (20.3-27.3)	6.0 (3.0-9.5)	60.0 (59.8-61.3)
**Probiotic**	2.0 (0.8-3.0)	14.0 (13.0-18.0)	7.0 (3.8-12.3)	30.0 (28.0-31.3)	24.0 (18.5-27.0)	4.0 (3.0-9.0)	61.0 (59.0-62.3)
**MIST run 3**							
**Placebo**	2.0 (1.0-3.0)	13.5 (10.8-16.0)	7.0 (3.0-10.0)	30.5 (28.0-32.3)	24.5 (19.5-28.3)	5.0 (4.0-10.3)	60.5 (59.0-62.0)
**Probiotic**	2.0 (0.0-3.0)	14.5 (11.8-17.0)	7.0 (3.8-11.0)	31.5 (28.0-34.3)	22.5 (18.0-25.5)	4.0 (2.8-10.0)	61.0 (59.8-62.0)

*The number of tasks when subjects did not make any attempt to answer
Median and interquartile range (IQR 25 to 75) are reported
